# Supplementary material for: HIVprotI: an integrated web based platform for prediction and design of HIV proteins inhibitors
Source: J Cheminform. 2018 Mar 9;10:12. doi: 10.1186/s13321-018-0266-y (PMC5845081; doi:10.1186/s13321-018-0266-y)
Supplement: Supplementary file 2 — Additional file 2. Source code of HIVProtI web server. [file 13321_2018_266_MOESM2_ESM.zip › HIVprotI_Source-code/hivprotI/weka-3-6-6/doc/weka/core/Debug.html]

Debug


---


|  |  |  |
| --- | --- | --- |
| **Overview** \| **Package** \| **Class** \| **Tree** \| **Deprecated** \| **Index** \| **Help** \| **Weka's home** | |  |
| **PREV CLASS**   **NEXT CLASS** | **FRAMES**    **NO FRAMES**     **All Classes** |  |
| SUMMARY: NESTED \| FIELD \| CONSTR \| METHOD | DETAIL: FIELD \| CONSTR \| METHOD |  |


---


## weka.core Class Debug

```
java.lang.Object
  weka.core.Debug
```

**All Implemented Interfaces:**: java.io.Serializable, RevisionHandler

---

``` public class Debug extends java.lang.Object implements java.io.Serializable, RevisionHandler ```

A helper class for debug output, logging, clocking, etc.

**Version:**
:   $Revision: 7519 $

**Author:**
:   fracpete (fracpete at waikato dot ac dot nz)

**See Also:**: Serialized Form

---

| **Nested Class Summary** | |
| --- | --- |
| `static class` | `Debug.Clock`             A little helper class for clocking and outputting times. |
| `static class` | `Debug.DBO`             contains debug methods |
| `static class` | `Debug.Log`             A helper class for logging stuff. |
| `static class` | `Debug.Random`             This extended Random class enables one to print the generated random numbers etc., before they are returned. |
| `static class` | `Debug.SimpleLog`             A little, simple helper class for logging stuff. |
| `static class` | `Debug.Timestamp`             A class that can be used for timestamps in files, The toString() method simply returns the associated Date object in a timestamp format. |


| **Field Summary** | |
| --- | --- |
| `static java.util.logging.Level` | `ALL`             the log level All |
| `static java.util.logging.Level` | `CONFIG`             the log level Vonfig |
| `static java.util.logging.Level` | `FINE`             the log level Fine |
| `static java.util.logging.Level` | `FINER`             the log level Finer |
| `static java.util.logging.Level` | `FINEST`             the log level Finest |
| `static java.util.logging.Level` | `INFO`             the log level Info |
| `static java.util.logging.Level` | `OFF`             the log level Off - i.e., no logging |
| `static java.util.logging.Level` | `SEVERE`             the log level Severe |
| `static java.util.logging.Level` | `WARNING`             the log level Warning |


**Constructor Summary**

`Debug()`
  
          default constructor, prints only to stdout

`Debug(java.lang.String filename)`
  
          logs the output to the specified file (and stdout).

`Debug(java.lang.String filename,
int size,
int numFiles)`
  
          logs the output


| **Method Summary** | |
| --- | --- |
| `Debug.Clock` | `getClock()`             returns the instance of the Clock that is internally used |
| `static java.lang.String` | `getCurrentDir()`             returns the current working directory of the user |
| `boolean` | `getEnabled()`             returns whether the logging is enabled |
| `static java.lang.String` | `getHomeDir()`             returns the home directory of the user |
| `java.lang.String` | `getRevision()`             Returns the revision string. |
| `static java.lang.String` | `getTempDir()`             returns the system temp directory |
| `static java.lang.Object` | `loadFromFile(java.lang.String filename)`             deserializes the content of the file and returns it, null if an error occurred. |
| `void` | `log(java.util.logging.Level level, java.lang.String message)`             prints the given message with the specified level and an empty sourceclass |
| `void` | `log(java.util.logging.Level level, java.lang.String sourceclass, java.lang.String message)`             prints the given message with the specified level |
| `void` | `log(java.util.logging.Level level, java.lang.String sourceclass, java.lang.String sourcemethod, java.lang.String message)`             prints the given message with the specified level |
| `void` | `log(java.lang.String message)`             prints the given message with level INFO |
| `static Debug.Clock` | `newClock()`             returns a new instance of a clock |
| `static Debug.Log` | `newLog(java.lang.String filename, int size, int numFiles)`             returns a new Log instance |
| `static java.util.Random` | `newRandom()`             returns a default debug random object, with no particular seed and debugging enabled. |
| `static java.util.Random` | `newRandom(int seed)`             returns a debug random object with the specified seed and debugging enabled. |
| `static Debug.Timestamp` | `newTimestamp()`             returns a default timestamp for the current date/time |
| `static boolean` | `saveToFile(java.lang.String filename, java.lang.Object o)`             writes the serialized object to the speicified file |
| `void` | `setEnabled(boolean value)`             sets whether the logging is enabled or not |
| `void` | `startClock()`             starts the clock |
| `void` | `stopClock(java.lang.String message)`             stops the clock and prints the message associated with the time, but only if the logging is enabled. |
| `static java.util.logging.Level` | `stringToLevel(java.lang.String level)`             turns the string representing a level, e.g., "FINE" or "ALL" into the corresponding level (case-insensitive). |
| `static boolean` | `writeToFile(java.lang.String filename, java.lang.Object obj)`             Writes the given object to the specified file. |
| `static boolean` | `writeToFile(java.lang.String filename, java.lang.Object obj, boolean append)`             Writes the given object to the specified file. |
| `static boolean` | `writeToFile(java.lang.String filename, java.lang.String message)`             Writes the given message to the specified file. |
| `static boolean` | `writeToFile(java.lang.String filename, java.lang.String message, boolean append)`             Writes the given message to the specified file. |

**Methods inherited from class java.lang.Object**

`equals, getClass, hashCode, notify, notifyAll, toString, wait, wait, wait`

**Field Detail**

### ALL

```
public static final java.util.logging.Level ALL
```

:   the log level All

---


### CONFIG

```
public static final java.util.logging.Level CONFIG
```

:   the log level Vonfig

---


### FINE

```
public static final java.util.logging.Level FINE
```

:   the log level Fine

---


### FINER

```
public static final java.util.logging.Level FINER
```

:   the log level Finer

---


### FINEST

```
public static final java.util.logging.Level FINEST
```

:   the log level Finest

---


### INFO

```
public static final java.util.logging.Level INFO
```

:   the log level Info

---


### OFF

```
public static final java.util.logging.Level OFF
```

:   the log level Off - i.e., no logging

---


### SEVERE

```
public static final java.util.logging.Level SEVERE
```

:   the log level Severe

---


### WARNING

```
public static final java.util.logging.Level WARNING
```

:   the log level Warning


**Constructor Detail**

### Debug

```
public Debug()
```

:   default constructor, prints only to stdout

---


### Debug

```
public Debug(java.lang.String filename)
```

:   logs the output to the specified file (and stdout). Size is 1,000,000 bytes
    and 1 file.

    **Parameters:**: `filename` - the name of the log

---


### Debug

```
public Debug(java.lang.String filename,
             int size,
             int numFiles)
```

:   logs the output

    **Parameters:**: `filename` - the name of the log: `size` - the size of the files in bytes: `numFiles` - the number of files for rotating


**Method Detail**

### stringToLevel

```
public static java.util.logging.Level stringToLevel(java.lang.String level)
```

:   turns the string representing a level, e.g., "FINE" or "ALL" into
    the corresponding level (case-insensitive). The default is ALL.

    :   **Parameters:**: `level` - the string to return a level for **Returns:**: the corresponding level or the default

---


### newLog

```
public static Debug.Log newLog(java.lang.String filename,
                               int size,
                               int numFiles)
```

:   returns a new Log instance

    :   **Parameters:**: `filename` - the name of the log: `size` - the size of the files in bytes: `numFiles` - the number of files for rotating **Returns:**: the log instance

---


### log

```
public void log(java.lang.String message)
```

:   prints the given message with level INFO

    :   **Parameters:**: `message` - the message to print

---


### log

```
public void log(java.util.logging.Level level,
                java.lang.String message)
```

:   prints the given message with the specified level and an empty sourceclass

    :   **Parameters:**: `level` - the level of logging: `message` - the message to print

---


### log

```
public void log(java.util.logging.Level level,
                java.lang.String sourceclass,
                java.lang.String message)
```

:   prints the given message with the specified level

    :   **Parameters:**: `level` - the level of logging: `sourceclass` - the class that logs the message: `message` - the message to print

---


### log

```
public void log(java.util.logging.Level level,
                java.lang.String sourceclass,
                java.lang.String sourcemethod,
                java.lang.String message)
```

:   prints the given message with the specified level

    :   **Parameters:**: `level` - the level of logging: `sourceclass` - the class that logs the message: `sourcemethod` - the method that logs the message: `message` - the message to print

---


### setEnabled

```
public void setEnabled(boolean value)
```

:   sets whether the logging is enabled or not

    :   **Parameters:**: `value` - if true logging will be enabled

---


### getEnabled

```
public boolean getEnabled()
```

:   returns whether the logging is enabled

    :   **Returns:**: true if the logging is enabled

---


### newClock

```
public static Debug.Clock newClock()
```

:   returns a new instance of a clock

    :   **Returns:**: a new instance of a Clock

---


### getClock

```
public Debug.Clock getClock()
```

:   returns the instance of the Clock that is internally used

    :   **Returns:**: the clock that's being used

---


### startClock

```
public void startClock()
```

:   starts the clock

---


### stopClock

```
public void stopClock(java.lang.String message)
```

:   stops the clock and prints the message associated with the time, but only
    if the logging is enabled.

    :   **Parameters:**: `message` - the message to print **See Also:**: `getEnabled()`

---


### newRandom

```
public static java.util.Random newRandom()
```

:   returns a default debug random object, with no particular seed and
    debugging enabled.

    :   **Returns:**: a new instance of a Random object

---


### newRandom

```
public static java.util.Random newRandom(int seed)
```

:   returns a debug random object with the specified seed and debugging
    enabled.

    :   **Parameters:**: `seed` - the seed value **Returns:**: a new instance of a Random object

---


### newTimestamp

```
public static Debug.Timestamp newTimestamp()
```

:   returns a default timestamp for the current date/time

    :   **Returns:**: a new timestamp

---


### getTempDir

```
public static java.lang.String getTempDir()
```

:   returns the system temp directory

    :   **Returns:**: the temp directory

---


### getHomeDir

```
public static java.lang.String getHomeDir()
```

:   returns the home directory of the user

    :   **Returns:**: the user's home directory

---


### getCurrentDir

```
public static java.lang.String getCurrentDir()
```

:   returns the current working directory of the user

    :   **Returns:**: the user's current working directory

---


### writeToFile

```
public static boolean writeToFile(java.lang.String filename,
                                  java.lang.Object obj)
```

:   Writes the given object to the specified file. The string representation
    of the object is appended to the file.

    :   **Parameters:**: `filename` - the file to write to: `obj` - the object to write to the file **Returns:**: true if writing was successful

---


### writeToFile

```
public static boolean writeToFile(java.lang.String filename,
                                  java.lang.String message)
```

:   Writes the given message to the specified file. The message is appended
    to the file.

    :   **Parameters:**: `filename` - the file to write to: `message` - the message to write **Returns:**: true if writing was successful

---


### writeToFile

```
public static boolean writeToFile(java.lang.String filename,
                                  java.lang.Object obj,
                                  boolean append)
```

:   Writes the given object to the specified file. The string representation
    of the object is either appended or replaces the current content of the
    file.

    :   **Parameters:**: `filename` - the file to write to: `obj` - the object to write to the file: `append` - whether to append the message or not **Returns:**: true if writing was successful

---


### writeToFile

```
public static boolean writeToFile(java.lang.String filename,
                                  java.lang.String message,
                                  boolean append)
```

:   Writes the given message to the specified file. The message is either
    appended or replaces the current content of the file.

    :   **Parameters:**: `filename` - the file to write to: `message` - the message to write: `append` - whether to append the message or not **Returns:**: true if writing was successful

---


### saveToFile

```
public static boolean saveToFile(java.lang.String filename,
                                 java.lang.Object o)
```

:   writes the serialized object to the speicified file

    :   **Parameters:**: `filename` - the file to serialize the object to: `o` - the object to serialize **Returns:**: true if writing was successful

---


### loadFromFile

```
public static java.lang.Object loadFromFile(java.lang.String filename)
```

:   deserializes the content of the file and returns it, null if an error
    occurred.

    :   **Parameters:**: `filename` - the name of the file to deserialize **Returns:**: the deserialized content, null if problem occurred

---


### getRevision

```
public java.lang.String getRevision()
```

:   Returns the revision string.

    :   **Specified by:**: `getRevision` in interface `RevisionHandler`
    :   **Returns:**: the revision


---


|  |  |  |
| --- | --- | --- |
| **Overview** \| **Package** \| **Class** \| **Tree** \| **Deprecated** \| **Index** \| **Help** \| **Weka's home** | |  |
| **PREV CLASS**   **NEXT CLASS** | **FRAMES**    **NO FRAMES**     **All Classes** |  |
| SUMMARY: NESTED \| FIELD \| CONSTR \| METHOD | DETAIL: FIELD \| CONSTR \| METHOD |  |


---
